# DeepES: deep learning-based enzyme screening to identify orphan enzyme genes

**DOI:** 10.1093/bioinformatics/btaf053

**Published:** 2025-02-06

**Authors:** Keisuke Hirota, Felix Salim, Takuji Yamada

**Affiliations:** School of Life Science and Technology, Institute of Science Tokyo, Tokyo, 152-8550, Japan; School of Life Science and Technology, Institute of Science Tokyo, Tokyo, 152-8550, Japan; School of Life Science and Technology, Institute of Science Tokyo, Tokyo, 152-8550, Japan; Metagen, Inc., Yamagata, 997-0052, Japan; Metagen Therapeutics, Inc., Yamagata, 997-0052, Japan; digzyme, Inc., Tokyo, 105-0001, Japan

## Abstract

**Motivation:**

Progress in sequencing technology has led to determination of large numbers of protein sequences, and large enzyme databases are now available. Although many computational tools for enzyme annotation were developed, sequence information is unavailable for many enzymes, known as orphan enzymes. These orphan enzymes hinder sequence similarity-based functional annotation, leading gaps in understanding the association between sequences and enzymatic reactions.

**Results:**

Therefore, we developed DeepES, a deep learning-based tool for enzyme screening to identify orphan enzyme genes, focusing on biosynthetic gene clusters and reaction class. DeepES uses protein sequences as inputs and evaluates whether the input genes contain biosynthetic gene clusters of interest by integrating the outputs of the binary classifier for each reaction class. The validation results suggested that DeepES can capture functional similarity between protein sequences, and it can be implemented to explore orphan enzyme genes. By applying DeepES to 4744 metagenome-assembled genomes, we identified candidate genes for 236 orphan enzymes, including those involved in short-chain fatty acid production as a characteristic pathway in human gut bacteria.

**Availability and implementation:**

DeepES is available at https://github.com/yamada-lab/DeepES. Model weights and the candidate genes are available at Zenodo (https://doi.org/10.5281/zenodo.11123900).

## 1 Introduction

Progress in sequencing technology has yielded large numbers of protein sequences, including enzyme sequences. Although large enzyme databases, such as Kyoto Encyclopedia of Genes and Genomes (KEGG) (Kanehisa *et al.* 2021) and the BRENDA (Chang *et al.* 2021), are available, many enzymes registered in these databases lack sequence information. Enzymes with confirmed activity for which associated protein sequence information is lacking are termed orphan enzymes (Lespinet and Labedan 2005, Pouliot and Karp 2007). Reactions involving orphan enzymes involved in 20.8% of the 11 914 metabolic reactions described in the KEGG database (KEGG FTP Release 27 March 2023). Furthermore, at least 22.4% of enzymes with Enzyme Commission (EC) numbers are orphans (Sorokina *et al.* 2014). Leveraging genome-context information is useful for identifying orphan enzyme genes (Yamada *et al.* 2012); however, a computational approach for this identification has not been developed.

Orphan enzymes cannot be detected using sequence similarity-based functional annotation approaches, such as BLAST (Altschul *et al.* 1990), because their sequences are unknown. This issue severely limits biological function studies based on molecular data and creates gaps between the enormous sequence data available and the understanding of biological processes (Yamada *et al.* 2012, Zhang and Li 2023). For instance, diverse metabolic processes performed by the human gut microbiota, such as short-chain fatty acid production, influence gut inflammation, and cancer progression (Parada Venegas *et al.* 2019, Rebersek 2021, Liu *et al.* 2023); however, many of these reactions involve orphan enzymes (Shiroma *et al.* 2024). Therefore, an approach that assigns enzymatic activities to gene sequences without calculating sequence similarity is essential for understanding the role of orphan enzymes.

Functions can be predicted from a gene sequence independently of reference sequence databases. Recently, deep learning has enabled prediction of protein 3D structures (Jumper *et al.* 2021, Lin *et al.* 2023) and function classification from a protein sequence with high accuracy (Sureyya Rifaioglu *et al.* 2019, Cao and Shen 2021, Littmann *et al.* 2021, Sanderson *et al.* 2023). Most deep learning-based approaches focusing on enzymes, including DeepEC (Ryu *et al.* 2019), CLEAN (Yu *et al.* 2023), EnzBert (Buton *et al.* 2023), and DeepECtransformer (Kim *et al.* 2023), predict enzymatic activity based solely on a protein sequence in a powerful and efficient manner. EC numbers have also been used as enzymatic activity classes. The EC number, the most well-known enzyme classification system, describes enzymatic reactions hierarchically using four-digit numbers. Although the EC number is useful, the specified EC number requires complete description of the enzymatic activity. Therefore, it is incompatible with orphan enzymes, as the activities of these enzymes are often unclear. To overcome this limitation, we utilized Reaction Class (RClass), a classification system based on chemical transformation patterns for substrate and product pairs (Kotera *et al.* 2004, Muto *et al.* 2013). RClass can be used to classify incomplete reactions because classifications are defined from the fingerprint of a substrate–product pair, making this approach more appropriate than using EC numbers for orphan enzymes.

**Figure 1.**
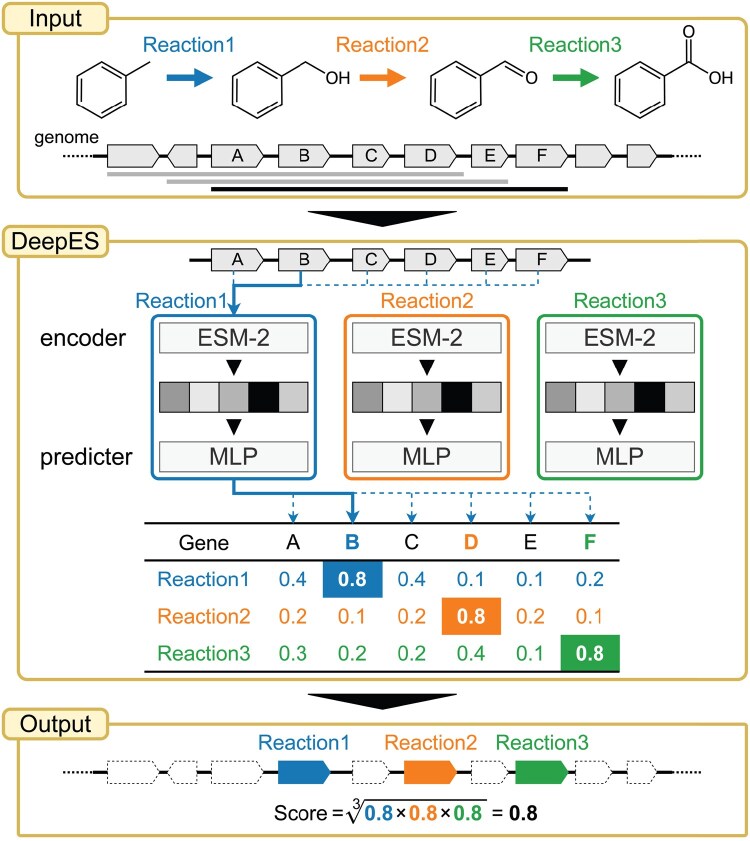
DeepES overview. The flow chart of exploring candidate genes for sequential enzymes using DeepES (our proposed method) consists of a three-step process: DeepES (1) retrieves protein sequences of successive genes in the genome from the input gene list, (2) infers independently the probability that each gene corresponds to each enzyme using the RClass classifiers for the enzyme reactions of interest, and (3) evaluates whether the sequential genes are likely to encode the enzymes for given successive chemical reactions based on a geometric mean of predicted probabilities. MLP, multilayer perceptron.

In this study, we developed DeepES, a deep learning-based framework focusing on sequential orphan enzymes using biosynthetic gene clusters (BGCs) for *in silico* orphan enzyme screening using RClass (Fig. 1). DeepES utilizes ESM-2, a pre-trained large language model that convert protein sequences to vector representations (Lin *et al.* 2023). We show that DeepES can identify candidate genes for orphan enzymes. DeepES consists of RClass binary classifiers, which predict whether the input genes correspond to each RClass, and the outputs of each model are integrated to evaluate whether the input genes contain the BGC, including an orphan enzyme of interest. Subsequently, we focused on human gut bacteria and explored candidate genes from 4744 metagenome-assembled genomes (MAGs) for 236 orphan enzymes, including enzymatic reactions for short-chain fatty acid production, as characteristic pathways of human gut bacteria.

## 2 Materials and methods

### 2.1 RClass

RClass is a fingerprint-based reaction classification schema and KEGG RCLASS database entry (Kotera *et al.* 2004, Muto *et al.* 2013). The KEGG RCLASS database contains all chemical transformation patterns associated with all reactions in the KEGG REACTION database. Each RClass represents the chemical transformation pattern of a substrate–product pair, and one reaction is often annotated with multiple RClasses. RClass allows a more abstract and automatic classification than the EC number does, and thus, can be applied to a wider range of enzymatic reactions. However, RClass is also an imbalanced classification, with only 684 of the 1600 RClasses accounting for 96% of the data (Supplementary Fig. S1 and Table S1).

### 2.2 Machine learning model development

#### 2.2.1 Dataset

We used prokaryotic gene sequences and RClass data retrieved from the KEGG database (KEGG FTP Release 27 March 2023) for the training, validation, and testing of machine learning (ML) models. KEGG Orthology (KO) is a dataset containing the corresponding enzymatic reactions from a gene to a protein orthology. Therefore, a dataset for ML models was constructed by pairing gene sequences and RClass using KO; the data included 4 413 823 cases. Protein sequences of more than 4000 amino acids or those containing “J” (leucine or isoleucine) were removed from the datasets because of the computational resources and specifications of ESM-2. The final dataset consisted of 4 413 788 prokaryotic gene protein sequences and RClass pairs split into 1 896 609 training data points and 2 517 179 test data points as of 15 May 2017.

#### 2.2.2 ML model development

RClass prediction is a multi-label classification task because several RClasses can be annotated for an enzyme reaction. Therefore, the ML models constituting DeepES were implemented as binary classifiers for each RClass. Each ML model consists of ESM-2 (Lin *et al.* 2023), a multilayer perceptron, and a simple feedforward fully connected neural network; i.e. the first half embeds the input gene sequence into a vector representation, and the second half predicts the probability that the input gene corresponds to the RClass of interest based on its embedding vector (Supplementary Fig. S2). We used ESM-2 with the specific version esm2_t33_650M_UR50D and froze its parameters. We also adopted a weighted loss function based on the class weight to address the class imbalance of RClass. If the enzyme reaction had multiple RClasses, the geometric mean of the model output corresponding to each RClass was calculated as the predicted probability, which indicated whether a given protein sequence encoded the enzyme.

#### 2.2.3 Hyperparameter tuning

To determine the optimal hyperparameters, we performed a grid search with 10-fold cross-validation on the training data and compared the area under the precision–recall curve (AUPRC). The hyperparameters included the number of epochs, hidden layer size, learning rate, and dropout rate (Table 1). We performed hyperparameter optimization of the multilayer perceptron for 100 RClass with over 1000 data points because the training data contained approximately 1500 RClasses.

**Table 1.** Hyperparameters.

Hyperparameters	Values	Selected values
Epoch	1, 5, 10, 20	20
Hidden layer size	64, 128, 256	64
Learning rate	1e−2, 5e−3, 1e−3	1e−3
Dropout rate	0.1, 0.25, 0.5	0.1

### 2.3 Validation in low sequence homology environments

#### 2.3.1 Dataset

To test the ability of our ML models to predict enzymatic functions that were difficult to annotate using BLAST, we constructed a small validation dataset with limited sequence homology. Specifically, we conducted sequence homology clustering of the test dataset using MMseqs2 (Steinegger and Söding 2017) with an identity threshold of 0.3 and the other parameters set to default values. After that, we created a small non-redundant validation dataset by picking a single representative sequence per cluster.

#### 2.3.2 Validation process

We repeated the steps of (1) constructing the training and test datasets, (2) training the ML models, and (3) evaluating ML model performance in a leave-one-out cross-validation manner using the validation dataset (Supplementary Fig. S3). Specifically, we chose one sequence (query sequence) and performed a homology search for the remainder of the dataset using BLAST (default) with the query sequence. We constructed a training dataset that excluded the hit sequences, and a test dataset by combining the query sequence data and negative data, which were 20 randomly selected non-enzyme genes from *Escherichia coli* K-12 MG1655. We trained the ML models and then evaluated their performance using the datasets. These procedures enabled us to calculate precision and evaluate whether the ML model output high predicted probabilities for positive examples and low predicted probabilities for negative examples. We also evaluated the reliability of the results of binary classification with a threshold for the predicted probability.

### 2.4 Biosynthetic gene cluster detection test

#### 2.4.1 DeepES framework

In bacterial, fungal, and plant genomes, groups of genes encoding enzymes, transporters, and regulators associated with a series of chemical reactions are often located close to each other in the genome; these gene are defined as BGCs, and are important for understanding metabolism in cellular systems (Medema *et al.* 2015, Kautsar *et al.* 2021). Furthermore, leveraging a combination of metabolic pathway adjacency and genomic neighborhood information can be used to obtain candidate genes of orphan enzyme genes (Yamada *et al.* 2012). Based on these findings, we developed DeepES, a deep learning-based framework focusing on BGCs for *in silico* orphan enzyme screening (Fig. 1). DeepES assumes that orphan enzyme genes constitute BGCs and aims to efficiently detect candidate genes. This framework takes a list of protein sequences of contiguous genes as input and repeats their evaluation using RClass classifiers corresponding to a series of enzyme reactions of interest. Subsequently, the set of contiguous genes slides the frame one by one from one end of the input gene list to the other and outputs a score representing the predicted probability that the BGC of interest falls within the range for each frame. The score for genes in the fixed scope of the input gene set was calculated by acquiring the highest predicted probability for each enzyme without gene overlap and calculating their geometric mean. For simplicity, the length of the contiguous reactions was 3, and the range of continuous genes evaluated was 10.

#### 2.4.2 Comparison to CLEAN

To compare DeepES with existing approaches, we performed BGC detection using CLEAN, the state-of-the-art EC number prediction tool (Yu *et al.* 2023). Specifically, we converted the EC numbers predicted by CLEAN (“max-separation” algorithm) into RClass using the KEGG API (Release 27 March 2023). We then used the converted results to detect BGCs by evaluating contiguous gene clusters, as in DeepES. However, the BGC score is binary because the output of CLEAN is not a predicted probability, unlike DeepES.

#### 2.4.3 Validation dataset

To validate the above BGC-based approach, 70 BGCs from the KEGG MODULE (KEGG FTP Release 27 March 2023) were used as pseudo-orphan enzymes. By preliminarily removing the KOs comprising these BGCs during training, we tested whether the proposed method could detect the candidate genes for pseudo-orphan enzymes. The 70 BGCs comprise a subset of the metabolic pathways in the KEGG MODULE that are detectable in this DeepES setting; i.e. a group of 10 successive genes in the genome containing three enzymes corresponding to three sequential enzyme reactions in the metabolic pathway are present in the test data (Supplementary Table S2). For simplicity, we excluded BGCs showing non one-to-one correspondence between enzyme genes and enzyme reactions.

#### 2.4.4 Real orphan enzymes dataset

We applied DeepES to actual orphan enzymes to identify genes encoding an orphan enzyme. We specifically searched for candidate genes from 4744 representative MAGs of human gut bacteria obtained from MGnify (Unified Human Gastrointestinal Genome v2.0.1) (Richardson *et al.* 2023) for 332 orphan enzymes in EnteroPathway, the metabolic pathway database of the human gut microbiota (Shiroma *et al.* 2024). A total of 1235 orphan enzymes was collected as enzyme reactions that were not associated with sequence information based on the correspondence of each entry with KEGG or UniProt provided by EnteroPathway, and 332 enzymes were selected as DeepES-applicable; i.e. 332 orphan enzymes were included in three consecutive enzyme reactions with RClass (Supplementary Table S3). We annotated RClass using E-zyme2 (Moriya *et al.* 2016) for enzymatic reactions in EnteroPathway when the RClass was not available on KEGG. Proteins with over 4000 residues or containing “J” (leucine or isoleucine) residue were excluded from the dataset because of limitations related to computational resources and specifications of ESM-2 when training and using the RClass classifiers.

## 3 Results

### 3.1 ML model development

**Figure 2.**
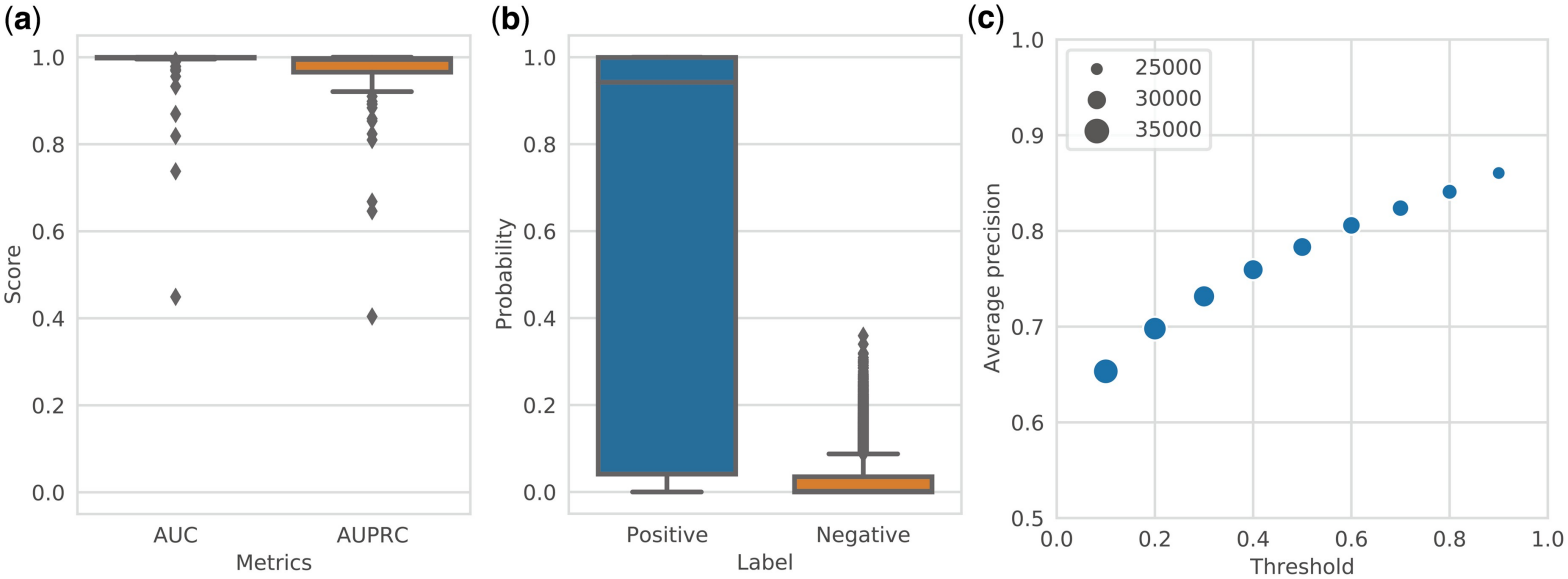
Performance of RClass binary classifier. (a) Predictive performance of the RClass binary classifiers on the test data (100 RClasses) evaluated by area under the receiver operating characteristic curve (AUC) and area under the precision-recall curve (AUPRC). (b) Distribution of predicted probabilities output by RClass binary classifiers in validation using BLAST. “Positive” and “negative” are output for query sequences and negative data, respectively. (c) Relationship of precision and threshold to predicted probability in validation using BLAST. The marker size represents the number of trials where precision could be calculated in leave-one-out cross-validation.

After a grid search with 10-fold cross-validation, we selected the best hyperparameters by measuring the average AUPRC (Table 1, Supplementary Table S1) and evaluated the performance of the ML models using the test dataset. For the best hyperparameters, the RClass binary classifiers exhibited a high AUC and AUPRC on the test dataset (Fig. 2a).

We next evaluated the predictive power of the ML models compared with that of BLAST by filtering the training dataset using BLAST as a leave-one-out cross-validation method. Although similar sequences in the test data were removed from the training dataset, the ML models provided high probabilities of predicting the correct RClass. The ML models showed high precision and appeared to clearly distinguish between positive and negative examples (Fig. 2b). Increasing the threshold for the predicted probability to 0.9 led to an average precision of 0.86, supporting the reliability of the upper end of the classifier's prediction results (Fig. 2c).

### 3.2 DeepES performance test

We treated known enzymes as orphan enzymes by removing the corresponding genes from the training dataset and tested whether DeepES could detect candidate genes for these enzymes. If the KO of an enzyme was removed from the training data and treated as an orphan enzyme, the positive examples for the RClass, which is the label for that enzyme, were sometimes missing from the training data, and the classifier could not be built. When the enzyme corresponding to the middle of three consecutive enzyme reactions was treated as an orphan enzyme, 36 of 70 were testable (hereafter referred to as “one orphan”); when all three were treated as orphan enzymes, 19 of 70 were testable (hereafter referred to as “all orphans”) (Supplementary Table S2).

**Figure 3.**
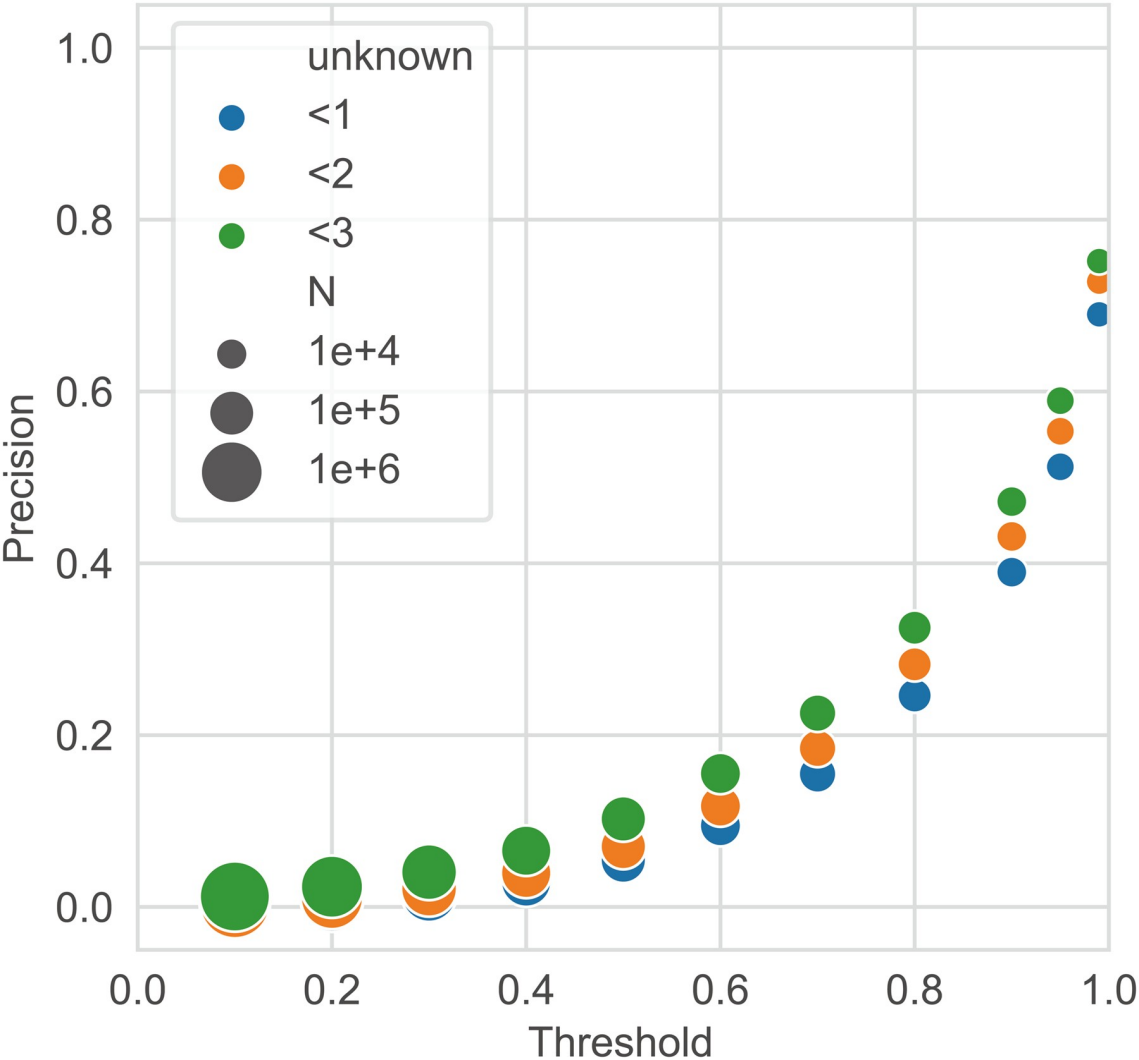
Validating DeepES on biosynthetic gene clusters (BGCs) with all orphans. Precision was calculated by summarizing the results of the DeepES predictions when the BGCs were selected from the 19 BGCs (all orphans) such that there was no overlap with the KEGG module, and the number of correct cases was maximized. The marker color indicates the number of hypothetical proteins that can be included in the genes hit by DeepES, and the marker size represents the number of outputs predicted as positive.

The results of applying DeepES to 36 BGCs (one orphan) and 19 BGCs (all orphans) showed that DeepES was able to detect the correct genes for the masked enzymes except for some BGCs (Supplementary Fig. S4 and Table S2). We selected the BGC with the largest number of positive cases per module to prevent duplication when evaluating enzymes and focused on those results (Fig. 3, Supplementary Table S2). Precision improved as the threshold for the DeepES output was increased; when the threshold was set to 0.99, the precision reached 0.690. Thus, approximately 70% of the scores above 0.99 in validation completely detected the three orphan enzyme genes, suggesting that the DeepES predictions with high scores are reliable. When all three genes selected by DeepES were hypothetical proteins, we excluded them from precision calculations because of evaluation difficulties. Furthermore, precision slightly improved when cases in which DeepES identified hypothetical proteins were considered. This result indicates that DeepES made an error and that a new gene for the enzyme could have been discovered. In addition, the results of the comparison with CLEAN suggest that DeepES is competitive with CLEAN for BGC detection (Supplementary Table S4).

### 3.3 Application to actual orphan enzymes

**Figure 4.**
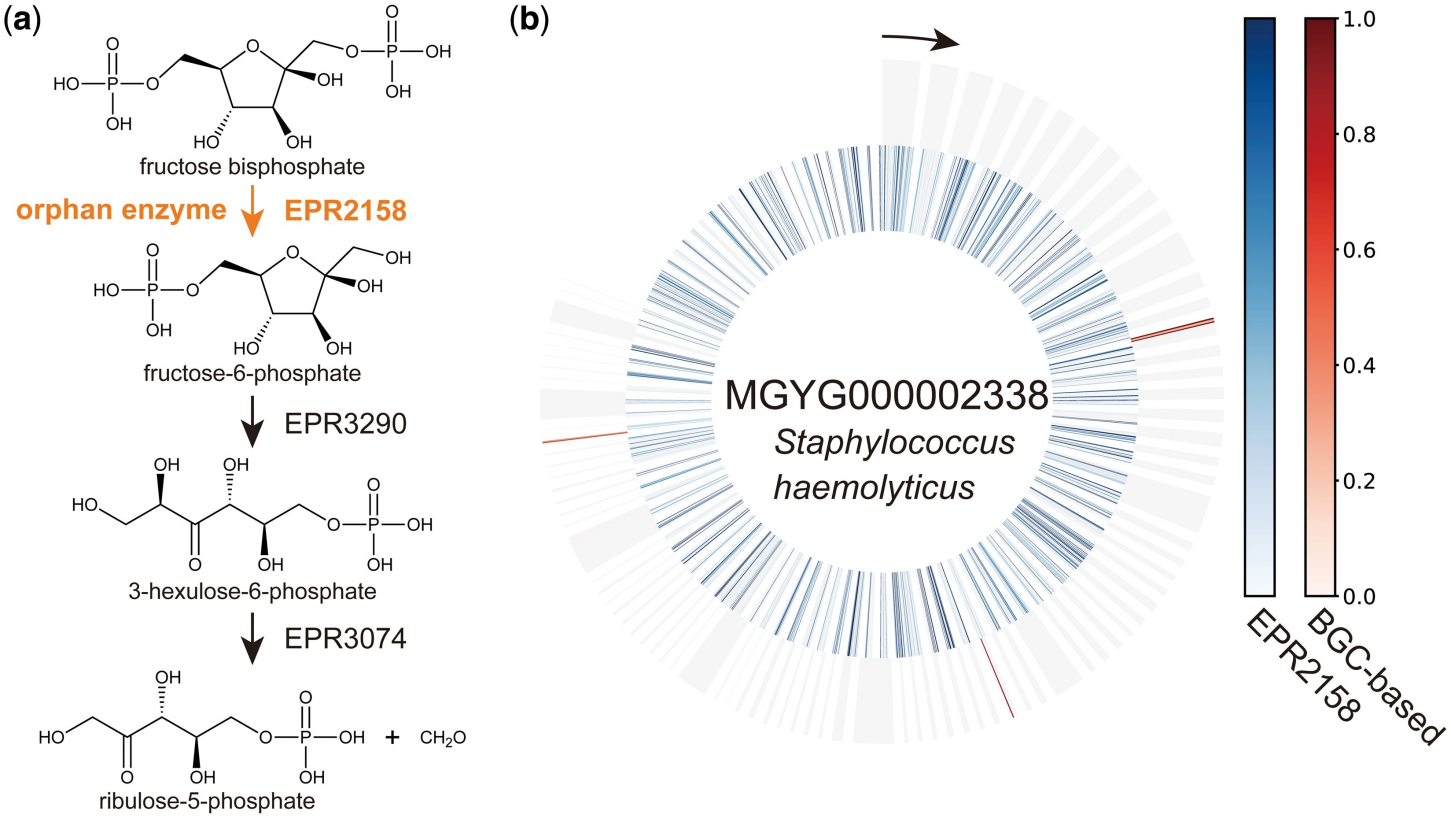
Example of orphan enzyme gene exploration using DeepES. (a) Example of part of a metabolic pathway involving enzymatic reactions by orphan enzymes. This part of energy metabolism by methane producers (EPM0948) and the first reaction (EPR2158) is performed by orphan enzymes. (b) Example of the result of applying DeepES to metagenome-assembled genomes (MAGs). The results for the MAG (MGYG000002338) with the highest score for the sequential enzyme reactions shown in (a) are illustrated. The inner circle represents the results of evaluating the orphan enzyme using only one RClass classifier, and the outer circle represents the results of evaluating the series of enzyme reactions using DeepES. The window size of DeepES is 10.

We focused on human intestinal bacteria and explored candidate genes for orphan enzymes using DeepES. We applied DeepES to 4744 representative MAGs from MGnify (Unified Human Gastrointestinal Genome v2.0.1) (Richardson *et al.* 2023) to explore candidate genes for 332 orphan enzymes to which DeepES is applicable and are associated with gut bacteria-specific metabolism in the EnteroPathway (Shiroma *et al.* 2024). DeepES identified candidate genes for orphan enzymes of interest, as in the BGC detection test. DeepES was used to annotate 3 102 987 genes with 236 orphan enzymes involved in metabolic pathways unique to human gut bacteria, including short-chain fatty acid production (Table 2, Supplementary Table S3). The BGC-based approach of DeepEC enabled us to narrow down candidate genes for orphan enzymes compared to evaluating a single gene and a single reaction (Fig. 4).

**Table 2.** Categories of pathway involving orphan enzymes with candidate genes.

Category		Count
Aromatic compounds	Shikimate biosynthetic pathway	31
	Others	75
Carbohydrates	Amino sugars and aminocyclitols	1
	Polysaccharides	10
	Sugar acids	1
	Sugar alcohols	1
Compounds derived from amino acids	Amino acids, peptides, and amines	8
Heterocyclic compounds	1-Ring heterocyclic compounds	1
	Other nitrogen heterocycles of natural origin	1
Isoprenoids	Diterpenoids	6
	Steroids	89
Organic chemicals	Triterpenoids	12
	Carboxylic acids	22

## 4 Discussions

We developed DeepES, a deep learning-based framework for enzyme screening focusing on BGCs and the RClass to identify orphan enzyme genes and demonstrated that the BGC-based approach may be useful for searching for orphan enzyme genes (Table 3). Evaluation of continuous genes inspired by BGCs is helpful for precise gene screening (Fig. 4). Unlike previous approaches, DeepES can screen orphan enzyme genes. Furthermore, the validation results using BLAST indicated that the ML model constituting DeepES is more sensitive than BLAST to functional similarity between sequences. DeepES also exhibited comparable performance to CLEAN, although some data leakage may have led to overestimated results as CLEAN was not retrained for the comparison (Supplementary Table S4).

**Table 3.** Comparison of DeepES with existing tools.

Characteristics	DeepES	DeepEC	CLEAN	EnzBERT	E-zyme 2	BLAST
Input	Protein sequences, reactions	Protein sequence	Protein sequence	Protein sequence	Substrate, product	Protein sequence
Output	Candidate genes	EC number	EC number	EC number	RClass	Sequence identity
Run independently of databases	○	○	○	○	×	×
Explore any sequence space	○	○	○	○	×	○
Can handle orphan enzymes	○	○	○	○	×	×
Can handle incorporate reactions	○	△	△	△	○	×
Compounds derived from amino acids	○	×	×	×	○	×
Leverage genome-context information	○	×	×	×	×	×

DeepES still has several limitations, including its representation of enzymatic activities and experimental validation. The use of RClass as a label for enzymatic activities allows ML models to predict enzymes without sufficient EC numbers, including orphan enzymes, whereas the class imbalance in the labels remains similar to that when using EC numbers. Although RClass is a fingerprint-based, high-quality reaction classification and can handle a wider range of enzyme reactions than can EC numbers, not all enzymatic reactions are annotated with RClass because RClass definition partially involves manual effort. Furthermore, RClass allows an approximate classification, which often leads to the assignment of the same RClass set to different enzyme reactions. Thus, the use of RClass can result in excessive candidate genes being hit, regardless of the performance of the RClass classifiers. However, no chemical reaction classification method is more automatic and more broadly applicable than, and of comparable quality to, RClass.

In this study, the default window size for BGC detection was set to 10, though this parameter is adjustable. The results from testing window sizes of 10, 20, and 30 indicated that 10 is acceptable in this case, but the most appropriate value would vary depending on the BGCs of interest (Supplementary Fig. S5 and Table S5).

As BGCs are found in plants and fungi, our proposed approach is expected to be potentially applicable to them, but this study focusses only on prokaryotes.

Many of the 236 orphan enzymes with candidate genes are involved in metabolic reactions associated with aromatic compounds or isoprenoids (Table 2). Some orphan enzyme reactions involved multiple pathways with different functions, such as EPR0399, which involved two functionally different pathways: organic chemicals (EPM0518) and aromatic compounds (EPM0665). Additionally, DeepES identified candidate genes for orphan enzymes involved in short-chain fatty acid production. Identifying genes corresponding to these enzymatic reactions may have many implications for studies of endogenous compounds, host–microbiota interactions (Rebersek 2021), and host metabolism (Gómez-Juaristi *et al.* 2018). MGYG000002338 (*Staphylococcus haemolyticus* strain: 864–1) scored highest in EPR2158, the enzymatic reaction involved in short-chain fatty acid production (Fig. 4). This predicted result may be promising, because some strains of *S. haemolyticus* have been reported to produce an acid from fructose (Takeuchi *et al.* 2005). The prediction results for the orphan enzyme genes require experimental validation.

In conclusion, we developed DeepES, a novel tool to identify orphan enzyme genes by leveraging BGCs information and fingerprint-based enzyme classification. DeepES identified candidate genes for 236 orphan enzymes associated with human gut bacteria, indicating the potential of DeepES to bridge the gap between sequence data and biological understanding.

## Supplementary Material

btaf053_Supplementary_Data

## Data Availability

No new data were generated in support of this research. DeepES is available at https://github.com/yamada-lab/DeepES. Model weights and the candidate genes are available at Zenodo (https://doi.org/10.5281/zenodo.11123900).

## Supplementary data

Conflict of interest: T.Y. is a founder of Metagen Inc., Metagen Therapeutics Inc., and digzyme Inc. Metagen Inc. focuses on the design and control of the gut environment for human health. Metagen Therapeutics Inc. focuses on drug discovery and development which utilizes microbiome science. All of the companies had no control over the interpretation, writing, or publication of this work. The terms of these arrangements are being managed by the Institute of Science in accordance with its conflict of interest policies.

## References

[btaf053-B1] Altschul SF , Gish W, Miller W et al Basic local alignment search tool. J Mol Biol 1990;215:403–10. 2231712 10.1016/S0022-2836(05)80360-2

[btaf053-B2] Buton N , Coste F, Le Cunff Y. Predicting enzymatic function of protein sequences with attention. Bioinformatics 2023;39:btad620. 37874958 10.1093/bioinformatics/btad620 PMC10612403

[btaf053-B3] Cao Y , Shen Y. TALE: transformer-based protein function annotation with joint sequence–label embedding. Bioinformatics 2021;37:2825–33. 33755048 10.1093/bioinformatics/btab198 PMC8479653

[btaf053-B4] Chang A , Jeske L, Ulbrich S et al BRENDA, the ELIXIR core data resource in 2021: new developments and updates. Nucleic Acids Res 2021;49:D498–508. 33211880 10.1093/nar/gkaa1025 PMC7779020

[btaf053-B5] Gómez-Juaristi M , Martínez-López S, Sarria B et al Absorption and metabolism of yerba mate phenolic compounds in humans. Food Chem 2018;240:1028–38. 28946219 10.1016/j.foodchem.2017.08.003

[btaf053-B6] Jumper J , Evans R, Pritzel A et al Highly accurate protein structure prediction with AlphaFold. Nature 2021;596:583–9. 34265844 10.1038/s41586-021-03819-2 PMC8371605

[btaf053-B7] Kanehisa M , Furumichi M, Sato Y et al KEGG: integrating viruses and cellular organisms. Nucleic Acids Res 2021;49:D545–51. 33125081 10.1093/nar/gkaa970 PMC7779016

[btaf053-B8] Kautsar SA , Blin K, Shaw S et al BiG-FAM: the biosynthetic gene cluster families database. Nucleic Acids Res 2021;49:D490–7. 33010170 10.1093/nar/gkaa812 PMC7778980

[btaf053-B9] Kim GB , Kim JY, Lee JA et al Functional annotation of enzyme-encoding genes using deep learning with transformer layers. Nat Commun 2023;14:7370. 37963869 10.1038/s41467-023-43216-z PMC10645960

[btaf053-B10] Kotera M , Okuno Y, Hattori M et al Computational assignment of the EC numbers for genomic-scale analysis of enzymatic reactions. J Am Chem Soc 2004;126:16487–98. 15600352 10.1021/ja0466457

[btaf053-B11] Lespinet O , Labedan B. Orphan enzymes? Science 2005;307:42. 10.1126/science.307.5706.42a 15637255

[btaf053-B12] Lin Z , Akin H, Rao R et al Evolutionary-scale prediction of atomic-level protein structure with a language model. Science 2023;379:1123–30. 36927031 10.1126/science.ade2574

[btaf053-B13] Littmann M , Heinzinger M, Dallago C et al Embeddings from deep learning transfer GO annotations beyond homology. Sci Rep 2021;11:1160. 33441905 10.1038/s41598-020-80786-0 PMC7806674

[btaf053-B14] Liu Y , Lau HC-H, Cheng WY et al Gut microbiome in colorectal cancer: clinical diagnosis and treatment. Genom Proteom Bioinform 2023;21:84–96. 10.1016/j.gpb.2022.07.002 PMC10372906 35914737

[btaf053-B15] Medema MH , Kottmann R, Yilmaz P et al Minimum information about a biosynthetic gene cluster. Nat Chem Biol 2015;11:625–31. 26284661 10.1038/nchembio.1890 PMC5714517

[btaf053-B16] Moriya Y , Yamada T, Okuda S et al Identification of enzyme genes using chemical structure alignments of substrate–product pairs. J Chem Inf Model 2016;56:510–6. 26822930 10.1021/acs.jcim.5b00216

[btaf053-B17] Muto A , Kotera M, Tokimatsu T et al Modular architecture of metabolic pathways revealed by conserved sequences of reactions. J Chem Inf Model 2013;53:613–22. 23384306 10.1021/ci3005379 PMC3632090

[btaf053-B18] Parada Venegas D , De la Fuente MK, Landskron G et al Short chain fatty acids (SCFAs)-mediated gut epithelial and immune regulation and its relevance for inflammatory bowel diseases. Front Immunol 2019;10:1486. 10.3389/fimmu.2019.00277 31316522 PMC6611342

[btaf053-B19] Pouliot Y , Karp PD. A survey of orphan enzyme activities. BMC Bioinformatics 2007;8:244. 17623104 10.1186/1471-2105-8-244 PMC1940265

[btaf053-B20] Rebersek M. Gut microbiome and its role in colorectal cancer. BMC Cancer 2021;21:1325. 34895176 10.1186/s12885-021-09054-2 PMC8666072

[btaf053-B21] Richardson L , Allen B, Baldi G et al MGnify: the microbiome sequence data analysis resource in 2023. Nucleic Acids Res 2023;51:D753–9. 36477304 10.1093/nar/gkac1080 PMC9825492

[btaf053-B22] Ryu JY , Kim HU, Lee SY. Deep learning enables high-quality and high-throughput prediction of enzyme commission numbers. Proc Natl Acad Sci U S A 2019;116:13996–4001. 31221760 10.1073/pnas.1821905116 PMC6628820

[btaf053-B23] Sanderson T , Bileschi ML, Belanger D et al ProteInfer, deep neural networks for protein functional inference. Elife 2023;12:e80942. 36847334 10.7554/eLife.80942 PMC10063232

[btaf053-B24] Shiroma H , Darzi Y, Terajima E et al Enteropathway: the metabolic pathway database for the human gut microbiota. Brief Bioinform 2024;25:bbae419. 39222063 10.1093/bib/bbae419 PMC11367760

[btaf053-B25] Sorokina M , Stam M, Médigue C et al Profiling the orphan enzymes. Biol Direct 2014;9:10. 24906382 10.1186/1745-6150-9-10 PMC4084501

[btaf053-B26] Steinegger M , Söding J. MMseqs2 enables sensitive protein sequence searching for the analysis of massive data sets. Nat Biotechnol 2017;35:1026–8. 29035372 10.1038/nbt.3988

[btaf053-B27] Sureyya Rifaioglu A , Doğan T, Jesus Martin M et al DEEPred: automated protein function prediction with multi-task feed-forward deep neural networks. Sci Rep 2019;9:7344. 31089211 10.1038/s41598-019-43708-3 PMC6517386

[btaf053-B28] Takeuchi F , Watanabe S, Baba T et al Whole-genome sequencing of *Staphylococcus haemolyticus* uncovers the extreme plasticity of its genome and the evolution of human-colonizing staphylococcal species. J Bacteriol 2005;187:7292–308. 16237012 10.1128/JB.187.21.7292-7308.2005 PMC1272970

[btaf053-B29] Yamada T , Waller AS, Raes J et al Prediction and identification of sequences coding for orphan enzymes using genomic and metagenomic neighbours. Mol Syst Biol 2012;8:581. 22569339 10.1038/msb.2012.13 PMC3377989

[btaf053-B30] Yu T , Cui H, Li JC et al Enzyme function prediction using contrastive learning. Science 2023;379:1358–63. 36996195 10.1126/science.adf2465

[btaf053-B31] Zhang Z , Li C. Enzyme annotation for orphan reactions and its applications in biomanufacturing. Green Chem Eng 2023;4:137–45.

