# Supplementary material for: DeepES: deep learning-based enzyme screening to identify orphan enzyme genes
Source: Bioinformatics. 2025 Feb 6;41(3):btaf053. doi: 10.1093/bioinformatics/btaf053 (PMC11881691; doi:10.1093/bioinformatics/btaf053)
Supplement: btaf053_Supplementary_Data [file btaf053_supplementary_data.zip › supplementary_materials/Suppelementary_materials.pdf]

Supplementary materials

DeepES: Deep learning-based enzyme screening to identify orphan enzyme genes

Keisuke Hirota[1], Felix Salim[1], Takuji Yamada[1,2,3,4]*

[1]School of Life Science and Technology, Tokyo Institute of Technology, Tokyo, Japan

[2]Metagen, Inc., Yamagata, Japan

[3]Metagen Therapeutics, Inc., Yamagata, Japan

[4]digzyme, Inc., Tokyo, Japan

*Corresponding author. School of Life Science and Technology, Tokyo Institute of Technology, Tokyo, Japan; E-mail: takuji@bio.titech.ac.jp

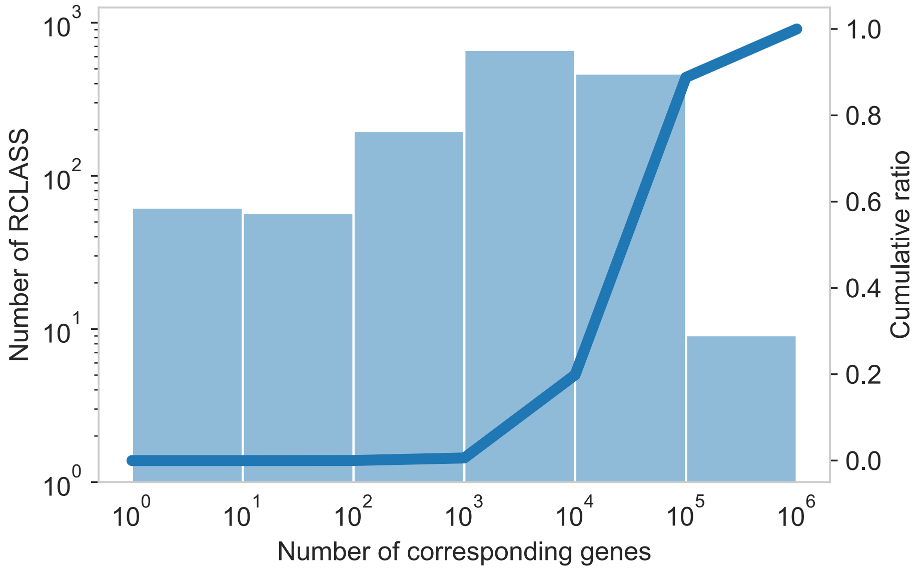

Figure S1 RClass distribution in the Kyoto Encyclopedia of Genes and Genomes (KEGG) dataset. The histogram represents the number of genes labeled with each RClass in the KEGG dataset. The line graph represents the KEGG dataset covered by the histogram bins.

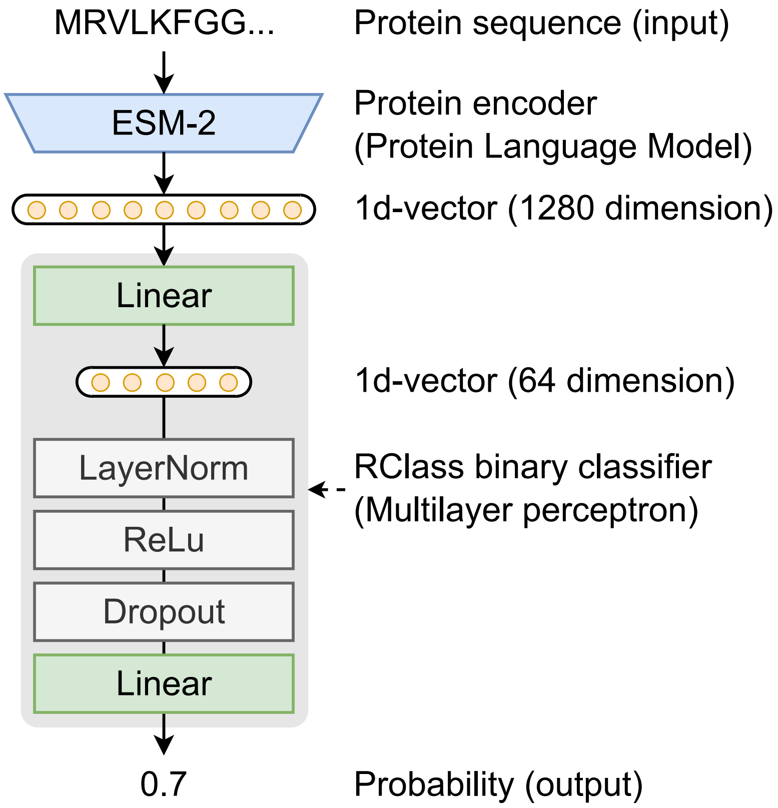

Figure S2 Architecture of the machine learning model constituting DeepES. Our model consists of ESM-2 and a 2-layer multilayer perceptron (MLP). To reduce overfitting, we applied layer normalization and dropout at a rate of 0.1 to MLP.

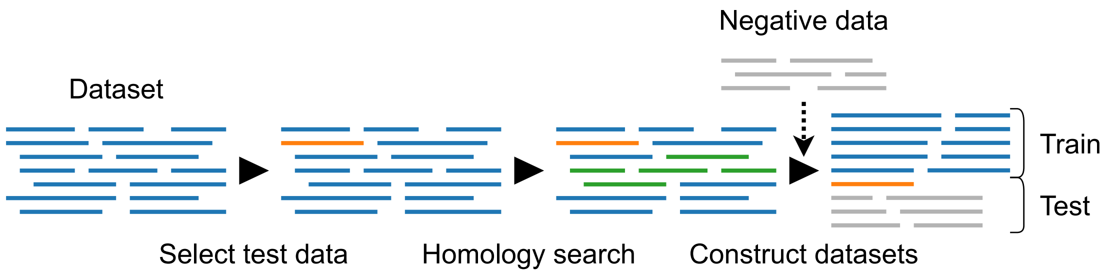

Figure S3 Workflow to construct validation datasets using BLAST. This process is repeated in leave-one-out cross-validation.

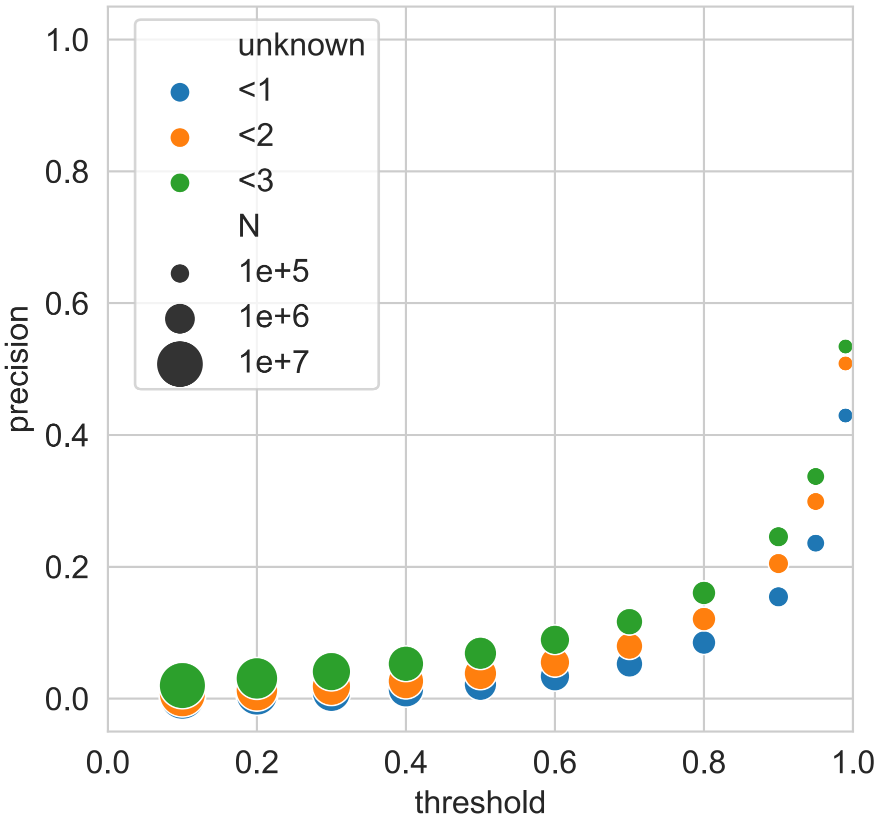

Figure S4 Validating DeepES on BGCs with one orphan. The precision was calculated by summarizing the results of the DeepES predictions for the 36 BGCs (one orphan). The marker color indicates how many hypothetical proteins can be included in the genes hit by DeepES, and the marker size represents the number of outputs predicted as positive.

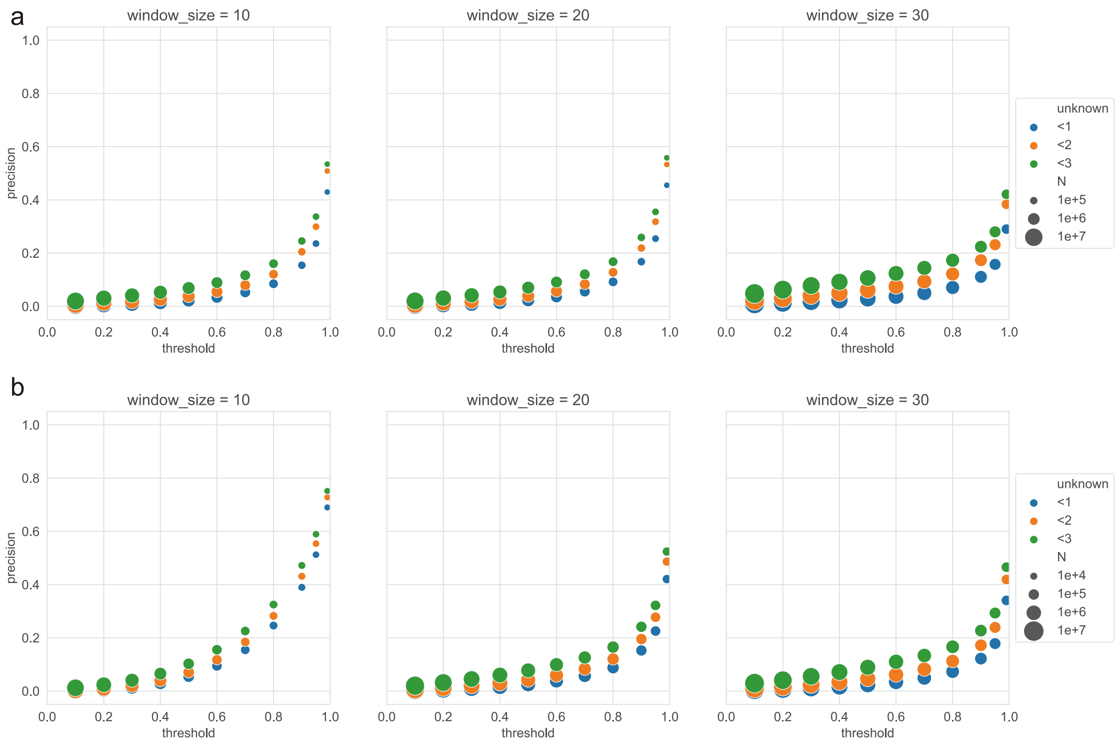

Figure S5 Comparison of results for different window sizes. For different window size values of 10, 20 and 30, we calculated the precision by summarizing the results of the DeepES predictions. The marker color indicates how many hypothetical proteins can be included in the genes hit by DeepES, and the marker size represents the number of outputs predicted as positive. (a) Results for the 36 BGCs (one orphan). (b) Results for the 19 BGCs (all orphans).
